# 

**Published:** 1846-06

**Authors:** 


### TO READERS AND CORRESPONDENTS.

In order that we may supply future subscribers with all the numbers of this vo'-ume of the Journal, we wish to preserve as many as possible of those first published. Post Masters, with uncalled for numbers in their offices, and Physicians not wishing to subscribe, or to retain such as they have received, will, therefore, confer a favor by returning them to the publishers.

To our Contributor, Dr. Stahl, we tender our thanks for two interesting articles; one of which, the "Case of the bite of a Mad Dog, and its Treatment," we publish in this number, the other, on the use of "Sulphate of Quinine in the congestive modifications of Scarlet Fever and Measles," will appear in our next.

We have received: –

The Practice of Surgery, by James Miller, F. R. S. E., F. R. C. S. E., Professor of Surgery in the University of Edinburgh, Surgeon to the Royal Infirmary, Author of the Principles of Surgery, &c. &c. Philadelphia: Lea & Blanchard. 1846. pp. 496. (From the Publishers. For sale by Brautigam & Keen, Chicago.)

A Clinical Introduction to the Practice of Auscultation, and other modes of Physical Diagnosis, intended to simplify the study of the diseases of the Lungs and Heart, by H. M. Hughes, M. D., Fellow of the Royal College of Physicians, Assistant Physician to Guy's Hospital, etc. Philadelphia: Lea & Blanchard, 1846. pp. 270. (From the Publishers. For sale by Brautigam & Keen, Chicago.)

Lectures on the Operations of Surgery, and on the diseases and accidents requiring operations, by Robert Liston, Esq., F. R. S. Senior Surgeon to the University College Hospital, and Professor of Clinical Surgery in the Hospital. With numerous additions by Thomas D. Mutter, M.D., Professor of Surgery in Jefferson Medical College, &c. &c. &c. Philadelphia: Lea & Blanchard. 1846. pp. 565. (From the Publishers. For sale by Brautigam and Keen, Chicago.)

On Diseases of the Liver, by George Budd, M.D., F. R. S., Professor of Medicine in King's College, London. and Fellow of Caius College, Cambridge, with colored plates and numerous wood cnts. Philadelphia: Lea & Blanchard. 1846. pp, 392. (From the Publishers. For sale by Brautigam & Keen, Chicago.)

Clinical Lectures on Surgery, delivered at St. George's Hospital by Sir Benjamin C. Brodie, Bart., V. P. R. S., Sergeant-Surgeon to the Queen, Surgeon in Ordinary to his Royal Highness Prince Albert, etc. etc. etc. Philadelphia: Lea & Blanchard. 1846. pp. 352. (From the Publishers. For sale by Brautigam & Keen, Chicago.)

The Influence of Tropical Climates on European Constitutions, by James Johnson, M. D., Physician to the late King, etc., and James Barnald Martin, Esq., late Presidency Surgeon, and Surgeon to the Native Hospital, Calcutta. From the Sixth London Edition, with notes by an American Physician. New York: Samuel S. & William Wood, 261 Pearl Street. 1846. pp. 624. (From the Publishers.)

A Manual of Chemistry, by Richard D. Hoblin, A. M., Oxon. Author of a Dictionary of Terms used in Medicine and the Collateral Sciences. New York: Samuel S. & William Wood, 261 Pearl Street 1846. pp. 335. (From the Publishers.)

Five Dissertions on Fever, by George Fordyce, M. D., F. R. S., Fellow of the Royal College of Physicians, &c. &c. Second American Edition with an introduction. Philadelphia,: Ed. Barrington & George D. Haskell. 1846. pp. 403. (From the Publishers.)

The above valuable works, for which we are much indebted to the Publishers, will hereafter receive due notice.

The Catalogue of Books on Medicine, Anatomy, Surgery, Midwifery, Chemistry, Agriculture, &c. &c. &c. for sale by Samuel S. & William Wood, 261, Pearl street, New York, gives the titles of a rare and most extensive assortment of works upon these subjects, and is worthy the attention of all who may wish to purchase.

We have received also—

The Boston Medical and Surgical Journal to May 27th, (in exchange.)

The New York Journal of Medicine and the Collateral Sciences, for May. (In Exchange.)

The New York Medical & Surgical Reporter, for April and May. (In Exchange.)

The Medical Examiner to May, with back Numbers. (In Exchange.)

The Bulletin of Medical Science for May. (In Exchange.)

Southern Medical & Surgical Journal for May. (In Exchange.)

The Western Lancet & Medical Library enlarged and improved. (In Exchange.)

The Western Journal of Medicine & Surgery for May. (In Exchange.)

The Missouri Medical & Surgical Journal for April. (In Exchange.)

The Buffalo Medical Journal for April. (In Exchange.)

Third Annual Report of the Managers of the State Lunatic Asylum, (New York) made to the Legislature, January 23d, 1846.

Twenty-fifth Annual Report of the Bloomingdale Asylum for the Insane, for the year 1845.

An Introductory Lecture delivered by G. S. Bedford, A. M., M. D., Professor of Midwifery in the New York University.

Catalogue of the Faculty and Students of the Medical Department of the University of the State of Missouri, for 1845-6.

Whole No. Students, 92. Graduates 29.

Also, Catalogue of the Trustees, Officers and Students of Indiana Medical College, (Medical Department of Laporte University.)

Whole No. of Students, 81, viz; Students in Chemistry, 10—Practitioners in and around Laporte, 5—Druggists, 2--Law Student 1=18—18 from 81 leaves 63 Medical Students—of these, 18 are set down as pupils of Prof. Meeker, and 17 as those of Prof. Richards, 18 & 17=35,—35 from 63 leaves 28 for the rest of the Faculty and the Profession at large.

---

### CONTRIBUTORS TO THE ILLINOIS & INDIANA MED. & SURG. JOUR.

A. H. Howland, M. D , Ottawa, Ill.
A. G. Henry, M. D., Pekin, Ill.
Edward Lewis, M. D., Jackson, Mich.
John McLain, M. D. Jackson, Mich.
Daniel Stahl, M. D., Quincy, Ill.
Edward Mead, M. D. Geneva, Ill.
Samuel G. Armor, M. D., Rockford, Ill.
Ira C. Bachus, M. D., Jackson, Mich.
J. G. Cornell, M. D., Spring Arbor, Mich.
G. N. Fitch, M. D., Logansport, Ind.
John F. Henry, M. D., Burlington, Iowa.

